# Medical negligence in healthcare organizations and its impact on patient safety and public health: a bibliometric study

**DOI:** 10.12688/f1000research.37448.1

**Published:** 2021-03-03

**Authors:** Saad Dahlawi, Ritesh G Menezes, Mohammad Ajmal Khan, Abu Waris, Saifullah, Mirza Muhammad Naseer

**Affiliations:** 1 Department of Environmental Health, College of Public Health, Imam Abdulrahman Bin Faisal University, Dammam 31441, P.O. Box 1982, Saudi Arabia; 2 Forensic Medicine Division, Department of Pathology, College of Medicine, Imam Abdulrahman Bin Faisal University, Dammam 31441, P.O. Box 1982, Saudi Arabia; 3 Deanship of Library Affairs, Imam Abdulrahman Bin Faisal University, Dammam 31441, P.O. Box 1982, Saudi Arabia

**Keywords:** Medical negligence, medical malpractice, medical errors, patient safety, bibliometric, scientometric

## Abstract

**Background**: Medical negligence is an increasing public health concern among healthcare providers worldwide as it affects patient safety. It poses a significant risk of patient injury, disease, disability, or death. The WHO has recognized deficiencies in patient safety as a global healthcare issue to be addressed. This study aimed to analyze various components of medical negligence research literature.

**Methods**: Bibliographic data visualizations tools like Biblioshiny (RStudio) and VOSviewer were used besides MS Excel to examine the types of documents, annual scientific production, top contributing authors and their impact, authorship patterns and collaboration, top contributing countries and organizations, most significant sources of publication, most cited documents, and most frequently used keywords. Bibliometric methods were used to analyze the bibliographic records of research output on medical negligence downloaded from the Web of Science Core Collection.

**Results**: The annual productivity of medical negligence research was increasing gradually. The most productive period for medical negligence research was 2011-2020. Bird contributed the highest number of publications to medical negligence literature while Brennan emerged as the highly cited author. Single-authored publications on medical negligence were not highly cited. The United States was the highest contributing country and the University of South Florida was the highest contributing organization while Harvard University was a highly cited organization. Nine out of the top ten contributing organizations were academic institutions and most of them belonged to the United States. The most important sources of publication on this topic were The Lancet and British Medical Journal. Localio *et al*. was the most important research article on medical negligence research.

**Conclusion**: Due to increasing attention on this topic, there was a sharp increase in the research output on medical negligence. This is of significance as the WHO set in motion a patient safety program almost two decades ago.

## Introduction

Medical negligence (also known as medical malpractice, medical errors, tort system) is an increasing public health concern among healthcare providers worldwide. The most comprehensive definition is “an act of omission or commission in planning or execution that contributes or could contribute to an unintended result” ( Grober & Bohnen, 2005; Thavarajah, Saranya & Priya, 2019). Medical negligence occurs when a healthcare professional selects the wrong method or procedure or improperly executes an appropriate method to treat or diagnose the patient ( Chukwuneke, 2015). There is no clear definition of medical negligence due to lack of nomenclature, overlapping of definitions, and lack of a standardized method to measure it ( Rodziewicz, Houseman & Hipskind, 2020).

All clinical practitioners and healthcare providers (e.g. physicians, nurses, medical technicians, paramedics, and other healthcare professionals) are responsible for any mistakes that could lead to medical negligence. There are several areas where medical negligence can arise, such as technical errors during surgical procedures, misdiagnosis of the disease, or prescribing the wrong medicine or incorrect dose ( Tariq, Vashisht, Sinha & Scherbak, 2020). These practices pose a significant risk of patient injury, disease, disability, or death. Subsequently, it may give rise to criminal and financial liabilities on hospitals and healthcare institutions ( He *et al.*, 2015; Ramanathan, 2014). Medical negligence lawsuits are focused on the medical professional's damage, injury, or failure to the patient. In general, medical negligence relief is given by means of penalties, i.e. monetary compensation ( Cheluvappa & Selvendran, 2020; Tumelty, 2020).

It is not easy to estimate the annual cost of liabilities and compensations on hospitals and public healthcare organizations. However, many studies show that this could be in billions of dollars per year. A study by the National Health Service in the United Kingdom estimated that the annual cost is around $1.20 billion ( Mathew, Asimacopoulos & Valentine, 2011). Medical negligence has been recognized for a long time by many researchers from different backgrounds. Several previous studies focused on the economic burden of medical negligence either on clinical practitioners as individuals or healthcare organizations as a management system. However, due to the complicity of this issue, it is not easy to estimate the exact cost of liabilities and compensations on doctors, hospitals, and healthcare organizations ( Mello, Chandra, Gawande, & Studdert, 2010).

The patient or the claimant has the right to file a lawsuit against clinicians by proving the following: the clinician owes a duty of care, there was a breach of that duty, and that breach caused the injury or damage ( Beran, Devereaux & Buchanan, 2020; Connelly & Serpell, 2020; Phillips, Thorne, Casey & Russo, 2021). Many previous studies focus on the estimation of annual cost and the financial liabilities on both the public and the private healthcare systems. The cost is not only the direct monetary expense that the doctors must pay but it also includes indirect costs such as physician’s time, stress, and loss of reputation ( Albano *et al.*, 2019). Furthermore, Wilson *et al.* (1995) reported that about 16% of 14000 hospitalization cases in Australia resulted in adverse disability due to medical negligence with legal implications. Moreover, doctors and medical professionals face lawsuits due to the cases filed against them due to negligen.

A study in Wuhan city in China performed 519 autopsies between 2004 and 2013 to evaluate medical negligence. The study showed that 36.6% of the death cases were due to medical malpractice ( He *et al.*, 2015). Every year, thousands of cases are filed in the courts against healthcare professionals due to tort cases ( Sohn, 2013). Despite the high occurrence of these cases, medical negligence is claimed to be under-reported in most healthcare settings ( Wu, 2000). It is, therefore, difficult to provide accurate statistics about medical negligence cases due to difficulties in analyzing and evaluating such type of data ( Jena, Seabury, Lakdawalla & Chandra, 2011; Rodziewicz, Houseman & Hipskind, 2020). There are many reasons for the limited availability of data related to medical negligence since not all hospitals have a clear policy for reporting every single medical error during routine medical procedures. Moreover, patients suffering from medical negligence may recover from damage and therefore may not be considered a medical negligence case thereafter.

As a result of criminal and financial liabilities arising due to medical negligence and the increasing demand to improve patient safety and quality care, there is an increased international focus on improving patient outcomes, safety, and quality of care that has led stakeholders, policymakers, and healthcare organizations to adopt standardized processes for evaluating healthcare organizations. Hospitals and healthcare organizations are now adopting standardized processes and an international accreditation system ( Alkhenizan & Shawb, 2011). The accreditation and certification system provide recommended guidelines and international standards to improve healthcare and patient safety in hospitals. The result is certification by an independent external auditor. Despite the national and international strategies for pushing hospitals and healthcare centers to be certified by recognized accreditation bodies, patient safety remains below the acceptable levels. Many studies proved that the effectiveness of such accreditation and certification is limited. Brubakk *et al.* (2015) claim that accreditation has little effect on patient outcomes, organizational culture, and reliability. Many other researchers argue that there was no convincing evidence on improving output quality and patient safety due to accreditation and certification ( Grepperud, 2015; Bogh, *et al.*, 2017).

Nevertheless, it is challenging to provide consistent solutions to eliminate or minimize recurrent events and work toward improving patient safety ( Oyebode 2013). Furthermore, it is essential that the governing bodies for the healthcare system should enforce hospitals to establish a litigation system by providing guidelines and steps to resolve the matter either by out of court settlement or a full court trial. This system should include effective policy and procedure to ensure high standards of effectiveness, transparency, and justice for all the involved parties ( Alkhenizan & Shafiq, 2018).

This research paper aimed to summarize the previous research done in this area and to determine the existing practice to control such issues. The trends of previously published research on this topic have been highlighted by emphasizing highly cited authors, international collaboration, keywords used, and analysis of future trends. Although several review articles on medical negligence have been published that summarize previous work ( Epstein, 2020; Connelly and Serpell, 2020; Birch and Todd, 2020), no bibliometric study has been published to date to analyze the research conducted in this field. In this study, a thorough evaluation of previously published literature on medical negligence and tort cases was conducted. Research output published in Web of Science were retrieved and analyzed to classify and determine the next steps and find out research gaps.

The bibliometric study is a quantitative analysis and statistical assessment to analyze the published articles using different parameters such as the leading authors and co-authors, keywords co-citations, document co-citations, institutes performance, international collaboration, etc. There is a notable growth trend in publication output along with more participation and collaboration of countries and institutes. The purpose of this type of analysis is to focus on the emerging trends and the knowledge structure on a topic. Using bibliometric tools, it is possible to generate easy to follow visual representations of complex correlations. This article provides a clear overview and general trends of research conducted on medical negligence over the last 67 years. It will highlight the highly cited publications and classify the existing literature into groups and clusters based on the latest developments and future trends.

### Objectives

The present study aimed to fulfill the following objectives relating to medical negligence research:

- To determine the types of documents
- To know the annual scientific production
- To find the top contributing authors and their impact
- To examine authorship and collaboration patterns
- To recognize top contributing countries and organizations
- To identify the most relevant sources of publication
- To discover the most cited documents
- To detect the most frequently used keywords

## Methods

### Source database and search query

The bibliographic records of research output on medical negligence research were downloaded from the Web of Science Core Collection (WOSCC) using the e-resources portal of Imam Abdulrahman Bin Faisal University (IAU). Web of Science (WOS) has been recognized as the most accurate and consistent indexing and abstracting database used by researchers worldwide and it has comprehensive coverage ( Birkle, *et al.*, 2020; Khan *et al.*, 2020; Tahira, Alias & Bakri, 2013). Data were downloaded on October 25th, 2020 using WOSCC category topic search (TS) with the following query:

TS= ("medical negligence") Refined by: [excluding] DOCUMENT TYPES: (NEWS ITEM OR NOTE OR MEETING ABSTRACT OR CORRECTION) Timespan: All years. Indexes: SCI-EXPANDED, SSCI, A&HCI, CPCI-S, CPCI-SSH, ESCI, CCR-EXPANDED, IC.

The present study was limited to publications on “medical negligence” indexed in the WOS database only; no other databases were used for bibliographic data. Therefore, the results of this study should be considered keeping in view the limitations of the study.

### Data selection

As compared to other search queries, we found the highest number of records for download with the search query above without applying any filter for time limit, country, or language. The total number of records downloaded and analyzed was 464. All publications relating to medical negligence were selected without any filter. Data were screened for duplication through Endnote Desktop X8 with matching options title, author, and year, which found zero duplicate records.

### Data analysis

Bibliometric methods were applied for the data analysis. Variables for which data analysis and visualization were performed included the following:

- **Annual scientific production**: Number of scientific publications produced in a year.
- **Top contributing authors and their impact**: Authors who contributed the most in the field of study and impact of their research in the field in terms of citations received.
- **Authorship and collaboration patterns**: Pattern of working of authors and how they collaborate with others to conduct the studies.
- **Top contributing countries and organizations**: Countries and organizations who contributed the most in the field of study.
- **Most relevant sources of publication**: Journals or sources where maximum number of documents were published relating to the field of study.
- **Most cited documents**: Documents which received highest number of citations.
- **Types of documents**: Forms of output of the documents like article, conference proceedings paper, review paper etc.
- **Frequently used keywords**: Keywords which were used more frequently by the authors.

### Analysis and visualization tools

Bibliographic data analysis and visualization tools Biblioshiny (RStudio, Version 1.2.5033) and VOSviewer (Version 1.6.13) were used in addition to MS Excel. Biblioshiny was used to determine the annual scientific production, top contributing authors and their impact, top contributing countries and organizations, most relevant sources of publication, most cited documents, and types of documents. MS Excel was used to determine the authorship and collaboration patterns while VOSviewer was used to visualize the frequently used keywords in medical negligence research.

## Results and discussion

Table 1 shows that the total number of documents was 464, out of which 304 documents (65.52%) were research articles, 66 documents (14.23%) reviews, and 49 documents (10.56%) were editorials. Research articles obtained the highest global citations with 3,374 citations (87.66%); reviews received 343 (8.92%) citations, and editorials received 108 (2.81%) citations. Overall, most of the documents were published as articles, which received more citations as compared to the other types of publications on the research topic.

**Table 1.** Documents type

Document type	Publications	Local citation score	Global citation score
Article	304	108	3,374
Review	66	13	343
Editorial	49	15	108
Letter	32	5	17
Proceedings	13	0	7
**Total**	**464**	**141**	**3,849**

Our results cover medical negligence research for 67 years. Table 2 displays the annual scientific productivity and citations per document on medical negligence research, and shows that annual productivity of medical negligence research has increased gradually. Research output was very low in the beginning with only nine research papers published from 1954 to 1980 with an accumulated percentage of 1.94%. Documents published from 1954 to 1980 did not receive any citations. However, research productivity significantly increased in the last two decades (2001-2020). From 2001 to 2010, 118 research documents were published, with an accumulated percentage of 25.43%. These publications received 9.09 citations per document. In the last decade (2011-2020), 216 research documents were published with an accumulated percentage of 46.55%, which received 3.70 citations per document. This shows that the most productive period for medical negligence research was 2011-2020, with about half of the total research output. Citation analysis showed that 70 documents were published on medical negligence research in 1991-2000, which received the highest number of citations (1,915) at a rate of 27.36 citations per document. Data revealed that no document was published in the years 1955 to 1957, 1959 to 1961, 1963 to 1975, and 1977 to 1978.

**Table 2.** Annual scientific productivity

Period	Total publications	Percentage	Cumulative percentage	Total citations	Total citations per document
1954-1960	4	0.86%	0.86%	0	0.00
1961-1970	2	0.43%	1.29%	0	0.00
1971-1980	3	0.65%	1.94%	0	0.00
1981-1990	51	10.99%	12.93%	62	1.22
1991-2000	70	15.09%	28.02%	1,915	27.36
2001-2010	118	25.43%	53.45%	1,073	9.09
2011-2020	216	46.55%	100.00%	799	3.70
**Total**	**464**	**100.00%**		**3,849**	**41.37**

Analysis of authors’ productivity revealed contributions from 974 authors to research on medical negligence. Table 3 presents the top ten authors who contributed to research on medical negligence along with their productivity. The analysis revealed that Bird S. (from MDA National, Sydney, Australia) contributed the highest number of documents (nine) to medical negligence research but received only 18 citations, with a citation impact of two. Samuels A. (from University of Southampton, United Kingdom) contributed five publications and received only three citations, while Brahams D. (from Lincoln's Inn, London, United Kingdom) contributed four publications and received only one citation. It demonstrates that most productive authors in the field of medical negligence are not highly cited. Brennan T. A. (from CVS, Woonsocket, United States) contributed only three publications but received the highest number of citations (754) with a citation impact of 251 per paper. Brennan was followed by Studdert D. M. and Fenn P. who contributed four documents each and received 275 and 86 citations, respectively.

**Table 3.** Top ten contributing authors and their impact

Authors (n = 974)	Affiliation	Country	Total publication (TP)	Total citations (TC)	Citation impact (TC/TP)	h_ index	Publication year start
Bird S	MDA National, Sydney	Australia	9	18	2	3	2007
Samuels A	University of Southampton	UK	5	3	1	1	1983
Brahams D	Lincoln's Inn, London,	UK	4	1	0	1	1981
Studdert D M	University of Melbourne	Australia	4	275	69	4	2000
Fenn P	University of Nottingham	UK	4	86	22	4	1994
Todd N V	Chris Moody Rehabilitation Centre	UK	4	8	2	2	2014
Popa T	RMIT University, Melbourne	Australia	4	4	1	1	2017
Tribe D M R	University of Hertfordshire, Hatfield	UK	4	4	1	1	1990
Brennan T A	CVS, Woonsocket	USA	3	754	251	3	1991
Bal B S	SINTX Technologies Corporation, Salt Lake City	USA	3	41	14	2	2012

**Figure 1.**
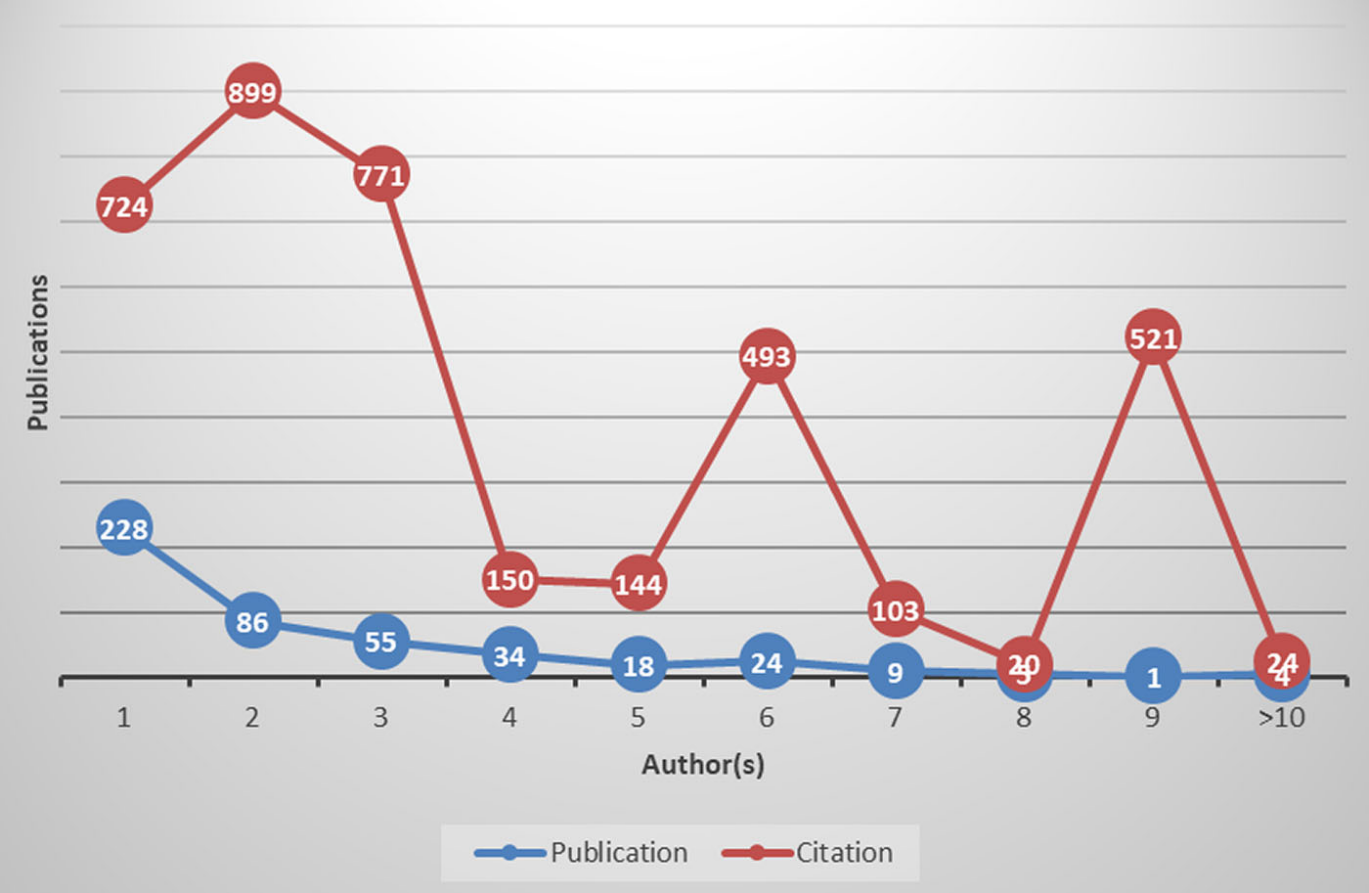
Authorship and collaboration pattern

Figure 1 describes the authorship pattern of medical negligence research. For authorship pattern, frequency of collaborating authors for each publication was analyzed, which ranged from a single author to 13 authors. There were 228 single-author publications, which obtained 724 citations while 86 publications were written by two authors and obtained 899 citations. It was revealed that single-authored publications on medical negligence were not highly cited. There were 55 publications with three authors and 34 publications with four authors, which obtained 771 and 150 citations, respectively. The frequencies of publications contributed by seven or more authors remained in single digits. Overall, the authorship pattern showed that most publications on medical negligence research (51%) were contributed by more than one author, which showed that authors contributing to medical negligence research were inclined towards collaborative research.

Country-wise analysis showed that 51 countries contributed 464 medical negligence research. The top ten contributing countries have been presented in Table 4. The United States was the highest contributing country with 96 occurrences. The publications affiliated with the United States obtained 1,735 citations, having a citation impact of 18.07 per paper. The United Kingdom was in second place with 83 occurrences and 625 citations, with a citation impact of 7.53 per paper. Australia was in the third position with 210 occurrences and 5.83 citation impact. Italy contributed only 17 documents but attained a good number of citations (119) and citation impact (7.00). Contributions from Japan and the Netherlands were in single digits, but the Netherlands obtained 77 citations with a citation impact of 12.83, which was the second-highest citation impact after the United States.

**Table 4.** Top ten contributing countries

Countries (n = 51)	Year	Total publications (TP)	Total citations (TC)	Citation Impact (TC/TP)
USA	1988	96	1,735	18.07
UK	1991	83	625	7.53
Australia	1998	36	210	5.83
India	1998	21	53	2.52
Peoples R China	2010	18	64	3.56
Italy	2000	17	119	7.00
Germany	1998	10	53	5.30
Canada	2005	10	42	4.20
Japan	2002	7	31	4.43
Netherlands	2003	6	77	12.83

Results of some previous studies were similar to this study, which exhibited that the United States was the most productive country in different areas of research, such as like Carpal Tunnel Syndrome, Coronavirus, Middle East Respiratory Syndrome (MERS), m-health, Sudden Infant Death Syndrome, and regulatory T-cell ( Bonilla-Aldana *et al.*, 2020; Danesh & Ghavidel, 2020; Menezes et al., 2020; Ram, 2019; Ram, 2020; Sweileh *et al.*, 2017; Wang *et al.*, 2016; Zongyi, *et al.*, 2016; Zyoud, 2016).

Analysis for organizations that participated in research on medical negligence revealed that 546 organizations contributed to the literature on the subject. In Table 5, the top ten contributing organizations have been presented along with their publications, citations, and citation impact. University of South Florida (United States) was the highest contributing organization with 16 publications, 58 citations, and 3.63 citation impact. Harvard University (United States) and the University of Melbourne (Australia) ranked second, each contributing nine publications. Harvard University obtained the highest number of citations (762) with 84.67 citation impact. This indicates that the most contributing organization was not widely cited in the area of medical negligence research. University of Padua (Italy) and Queensland University of Technology (Australia) ranked fourth, each contributing seven publications, but the University of Padua received more citations (29 with 4.14 citation impact) as compared to the Queensland University of Technology, which received only six citations with 0.86 citation impact. Other organizations included in the top ten contributed six publications each.

**Table 5.** Top ten contributing organizations

Rank	Organizations (n =546)	Country	Year of establishment	Total publications (TP)	Total citations (TC)	Citation Impact (TC/TP)
1	University of South Florida	USA	1956	16	58	3.63
2	Harvard University	USA	1636	9	762	84.67
2	University of Melbourne	Australia	1853	9	72	8.00
4	University of Padua	Italy	1222	7	29	4.14
4	Queensland University of Technology	Australia	1989	7	6	0.86
6	Oregon Health & Science University	USA	1887	6	39	6.50
6	Wayne State University	USA	1868	6	33	5.50
6	New York University	USA	1831	6	28	4.67
6	Children's Hospital of Philadelphia	USA	1855	6	20	3.33
6	Institute of Post Graduate Medical Education and Research	India	1957	6	6	1.00

Our analysis showed that nine out of the top ten contributing organizations were academic institutions and most of them (six) were based in the United States, which also confirmed the results of the country-wise analysis. Analogous results were exhibited during earlier studies, which found that universities contributed much of the research related to MERS, coronavirus, and regulatory T-cells ( Danesh & Ghavidel, 2020; Ram, 2020; Wang *et al.*, 2016; Zongyi, Dongying & Baifeng, 2016).

Publication source analysis disclosed that 464 documents on medical negligence research were published in 274 sources. Most of the documents were published in journals. In Table 6, the top 10 sources of publication have been presented. The top six sources published medical negligence literature in double-digits while less than ten documents were published by the remaining sources. The most important sources of publication for medical negligence research were *The Lancet* and *British Medical Journal*, which published 23 documents each and received 439 and 198 citations, respectively. These were followed by *Medicine Science* and *The Law* with 16 publications, which received nine citations. *Cardiology in The Young*, *Australian Family Practitioner*, and *Trial* contributed 10 publications each with 112 citations, 21 citations, and one citation, respectively.

**Table 6.** Top ten sources of publications

Rank	Source (n = 274)	Total publications	Total citations	Publications year start
1	*The Lancet*	23	439	1954
1	*British Medical Journal*	23	198	1984
3	*Medicine Science and The Law*	16	9	1983
4	*Cardiology in The Young*	10	112	2008
4	*Australian Family Physician*	10	21	2007
4	*Trial*	10	1	1979
7	*Clinical Orthopaedics and Related Research*	9	91	2005
7	*Medical Law Review*	9	33	2009
9	*Medical Journal of Australia*	7	92	1986
9	*Journal of Law and Medicine*	7	5	2015

The results of this study found that the most important sources of publication for medical negligence research were *The Lancet* and *British Medical Journal.* Some earlier studies discovered that the most favored journals for research on Carpal Tunnel Syndrome, Coronavirus, and MERS were the *Journal of Hand Surgery-American Volume*, and *Journal of Virology*, respectively ( Ram, 2019; Ram, 2020; Wang *et al.*, 2016; Zyoud, 2016).

In order to determine the status of research publications and the efficiency of researchers in any area of research, citations are a tool for comparative analysis of publications. The number of citations received specifies the standing of any publication in its field ( Ram, 2020). Table 7 presents the name of the first author, year of publication, and source of publication for the ten most cited research publications on medical negligence research. The study revealed that the research paper titled “Relation between Malpractice Claims and Adverse Events Due to Negligence — Results of the Harvard Medical Practice Study III” by Localio *et al.* published in *The New England Journal of Medicine (*doi:10.1056/NEJM199107253250405) was the most important research article on medical negligence research, which obtained 521 citations at a rate of 17.37 citations per year. The article titled “Why do people sue doctors? A study of patients and relatives taking legal action” by Vincent *et al.*, published in *The Lancet* (doi:10.1016/s0140-6736(94)93062-7) was the second most important research paper, which received 396 citations at a rate of 14.67 citations per year. The article titled “Assessments of Noneconomic Damage Awards in Medical Negligence: A Comparison of Jurors with Legal Professionals” by Vidmar, N. and Rice, Jeffrey J., published in *Iowa Law Rev*, volume 78, number 4, 1993, pp. 883-912, gained 48 citations at a rate of 1.71 citations per year.

**Table 7.** Top ten most cited documents

Rank	Documents (n = 464)	Total citations	Citation years	Total citations per year
1	Localio AR, 1991, New Engl J Med	521	30	17.37
2	Vincent C, 1994, Lancet	396	27	14.67
3	Kraman SS, 1999, Ann Intern Med	267	22	12.14
4	Studdert DM, 2000, Med Care	218	21	10.38
5	Summerton N, 1995, Brit Med J	105	26	4.04
6	Brady AP, 2017, Insights Imaging	70	4	17.50
7	Huycke LI, 1994, Ann Intern Med	69	27	2.56
8	Poonnoose PM, 2002, J Trauma	66	19	3.47
9	Hurwitz B, 2004, Brit Med J	55	17	3.24
10	Vidmar N, 1993, Iowa Law Rev	48	28	1.71

**Figure 2.**
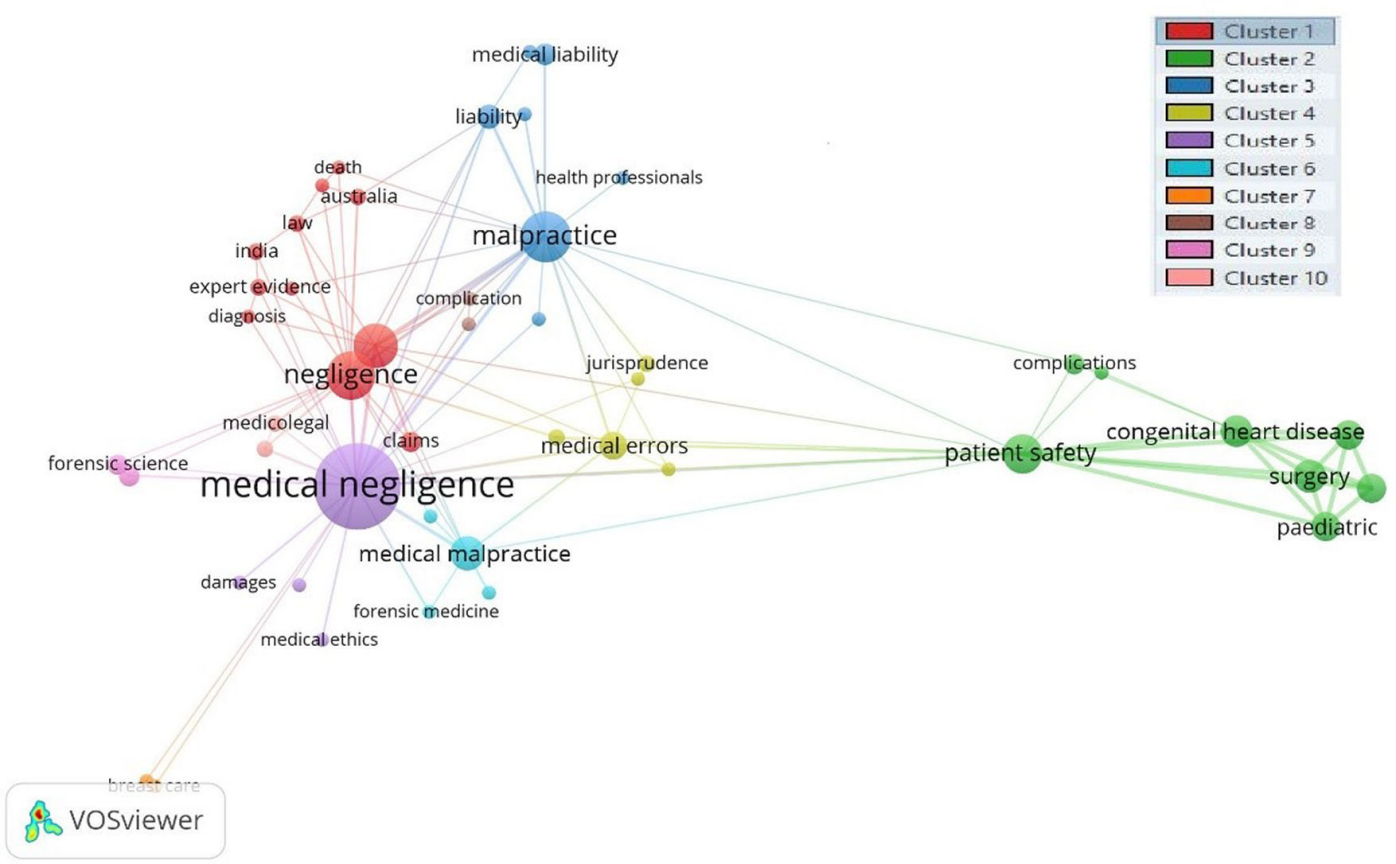
Author keywords.

Figure 2 displays the author keyword co-occurrence analysis of 798 keywords on medical negligence research using the full counting method. A threshold occurrence value of two was set for the analysis. In total, 92 keywords met the threshold value, but some items were not connected to each other. The largest set of connected items consisted of 85 items having ten clusters identified by different colors in Figure 2. The analysis revealed that 85 items, having 10 clusters, generated 279 links while the total link strength was 817. Most commonly keywords used by authors were medical negligence, malpractice, negligence, litigation, and patient safety.

## Conclusion

Based on the findings of this study, it can be understood that medical negligence research is published in various types of sources and a variety of output formats. We can conclude that research on medical negligence is getting the attention of the researchers, which has resulted in a sharp increase in the research output during the last two decades. This is of significance as the WHO set in motion a patient safety program almost two decades ago in the year 2004 recognizing deficiencies in patient safety as a global healthcare issue to be addressed ( WHO, 2004). Research on medical negligence is mostly concentrated in developed countries and contributing authors are inclined towards collaborative research. The study concludes that the accumulation of citations does not depend on the productivity of an author. It is recommended to replicate this study after ten years to observe future research trends in the field.

## Data availability

### Underlying data

Open Science Framework: Medical negligence in healthcare organizations and impacts on the patient safety: A bibliometric study, https://doi.org/10.17605/OSF.IO/DR3NZ ( Dahlawi *et al.*, 2021).

Data are available under the terms of the Creative Commons Zero “No rights reserved” data waiver (CC0 1.0 Public domain dedication).
