# Supplementary material for: Medical negligence in healthcare organizations and its impact on patient safety and public health: a bibliometric study
Source: F1000Res. 2021 Mar 3;10:174. [Version 1] doi: 10.12688/f1000research.37448.1 (PMC8156516; doi:10.12688/f1000research.37448.1)
Supplement: Peer review history

# Peer review: Reviewer response for version 1

**DOI:** 10.5256/f1000research.40511.r85963

**Published:** 2021-05-26

**Authors:** Rijen Shrestha

**Affiliations:** 1 Department of Forensic Medicine, Institute of Medicine, Tribhuvan University, Kathmandu, Nepal

This is an excellent bibliometric research on the output of research on medical negligence. The manuscript is well written, comprehensive and has relevant citations. This article does very well to inform the readers of the relevance of different types of publications, platforms, authorship etc.

 I found 'one' (1) typographical mistake - last word in fourth paragraph of Introduction (negligen - negligence). 

 Additionally, 'one' (1) minor edit that would provide clarity:

- Replace "clear definition" with "universally accepted definition" [last sentence of first paragraph in Introduction]

Finally, in the section on Results and Discussion, perhaps elaborate a little on the findings and discuss the relevance, interpretation of the findings as well.

 For example, Table 1 shows that articles are cited more frequently. Perhaps discuss in brief the relevance and inference of this finding. With 87.66% of citations, does it mean that it is being read 10X?

Is the work clearly and accurately presented and does it cite the current literature?

Yes

If applicable, is the statistical analysis and its interpretation appropriate?

Yes

Are all the source data underlying the results available to ensure full reproducibility?

Yes

Is the study design appropriate and is the work technically sound?

Yes

Are the conclusions drawn adequately supported by the results?

Yes

Are sufficient details of methods and analysis provided to allow replication by others?

Yes

Reviewer Expertise:

research methodology, statistical analysis, public health

I confirm that I have read this submission and believe that I have an appropriate level of expertise to confirm that it is of an acceptable scientific standard.

# Peer review: Reviewer response for version 1

**DOI:** 10.5256/f1000research.40511.r82341

**Published:** 2021-03-29

**Authors:** Ghulam Murtaza RAFIQUE

**Affiliations:** 1 Department of Information Management, University of Sargodha, Sargodha, Pakistan

The article "Medical negligence in healthcare organizations and its impact on patient safety and public health: a bibliometric study" addresses a topic in public health that is very timely. Medical negligence and its impact on patient safety and public health are areas that would be of interest to other professionals in the field of medical health. The article identifies why the topic was chosen, mentions how the area of medical negligence needed further research and study, and outlines the research objectives studied and the research design followed. The tables included in the article were clear to follow and were referenced properly in the article. I recommend its indexing in the journal.

 Overall, I enjoyed reading the article and think the findings of the study can shed light on the of literature on the topic and its contribution in the field.

Is the work clearly and accurately presented and does it cite the current literature?

Yes

If applicable, is the statistical analysis and its interpretation appropriate?

Yes

Are all the source data underlying the results available to ensure full reproducibility?

Yes

Is the study design appropriate and is the work technically sound?

Yes

Are the conclusions drawn adequately supported by the results?

Yes

Are sufficient details of methods and analysis provided to allow replication by others?

Yes

Reviewer Expertise:

Information behavior; Knowledge sharing; Bibliometric analysis; Information literacy

I confirm that I have read this submission and believe that I have an appropriate level of expertise to confirm that it is of an acceptable scientific standard.

# Response

**Published:** 2021-03-31

**Authors:** Saad Dahlawi

**Affiliations:** Imam Abdulrahman Bin Faisal University, Saudi Arabia

Dear reviewer

 Thanks for reading our manuscript and giving us your valuable feedback. We appreciate your quick and positive comments.

 Thank you very much.

 Saad Dahlawi

 The corresponding author
